# Paraffin-embedding for large volume bio-tissue

**DOI:** 10.1038/s41598-020-68876-5

**Published:** 2020-07-28

**Authors:** Ouyang Zhanmu, Xiaoying Yang, Hui Gong, Xiangning Li

**Affiliations:** 1 Britton Chance Center for Biomedical Photonics, Wuhan National Laboratory for Optoelectronics, MoE Key Laboratory for Biomedical Photonics, School of Engineering Sciences, Huazhong University of Science and Technology, Wuhan, 430074 China; 2 HUST-Suzhou Institute for Brainsmatics, JITRI Institute for Brainsmatics, Suzhou, 215123 China; 3 CAS Center for Excellence in Brain Science and Intelligence Technology, Chinese Academy of Science, Shanghai, 200031 China

**Keywords:** Immunohistochemistry, Biological techniques, Immunological techniques

## Abstract

Acquiring ultrahigh-resolution three-dimensional images of large-volume tissues non-human primate tissues was an enormous challenge. Given the preservation of structure and excellent sectioning property, formalin-fixed paraffin-embedding method had an enormous potential for three-dimensional reconstruction of fine structures, based on the very thin histological sections and optical images. However, maintaining the structure uniformly in large-volume tissues was difficult during the complex processes. In this study, we presented a detailed protocol for the whole mouse, rat, rabbit brains, and even for the macaque hemisphere. The entire protocol took about 2–30 days to complete for a large sample, including fixation, dehydration, clearing, wax immersion and embedding. In addition, it could be applied to other species and organs, while the embedding processes depended on the size and the type of organs. This method had wide applicability to serve as a baseline for further technique development.

## Introduction

As the research moved on, the comprehensive analysis of organs at single-cell resolution in the body had been one of the most fundamental challenges in biology and medicine, especially for nervous system. Many alternative tissue processing and imaging approaches had been proposed to acquire 3D images, including registering a series of serial thin sections^1,2^ and systematic optical imaging^3–6^. These methods had been applicable to mouse brains and allowed complete 3D visual examination at single-cell resolution^7–9^. With the neuroscience research moving along, non-human primates were increasingly used in studying of the mechanism underling the cognitive and the pathological mechanism of neural diseases. It was a formidable challenge to scale up the present imaging technologies to acquire the continuous structure information of these non-human primate brains. One of the main reasons was that the sheer size, companying with complex structure and variability among non-human primate brains brings much trouble in embedding. For example, the adult macaque brain was about 200–300 times bigger than mouse brain^10^, and it had a highly folded cerebral cortex. Therefore, researchers had to dissect these specimens of interest instead of dealing with the whole samples^11^. The dissection reduced the difficulty of operation, but it also disturbed the structure of these large samples. After all, an important premise to acquire the 3D structure of the whole specimen was preserving the integrity of specimens. Therefore, it was better embedding the whole specimen instead of dissection when combined with 3D imaging technologies.

To embed large specimens, the following were important criteria: the embedding medium could permeate into large tissues; embedded tissues had suitable hardness and stability for continuous sectioning within weeks at room temperature; the embedding method could provide single-cell resolution and compatible with partial histochemical staining. Considering these criteria, formalin-fixed and paraffin-embedding (FFPE) seemed to be particularly promising. There were four major reasons. First, FFPE could be widely used to various tissues such as brains, livers, lungs and tumors. This broad scope of application meant FFPE had a great compatibility of complex structure and variability among large tissues. Second, FFPE had an excellent property of sectioning. It produced smooth block surfaces and possess a well tissue morphology. Meanwhile, there were various optical imaging methods to incorporate FFPE, such as fluorescence imaging^12^, which was widely used in systematic optical imaging. These feathers mentioned above benefited it to reconstitute 3D images or combing with systematic optical imaging. Third, the chemistry and physics property of paraffin samples were very stable, and they could be stored at room temperature for months. It was convenient for long-term imaging. Finally, FFPE allowed semi-thin successive sections while leaving sections amenable to further characterization such as hematoxylin–eosin staining (H&E), hybridization in situ (ISH)^13^ or immunohistochemistry (IHC)^14^.

Although FFPE had a great potential in embedding large brains, traditional FFPE was usually used to embed small samples in 0.5–1 cm-thickness^15^. Clearing was a key factor, which limited the size of samples in paraffin embedding process. The traditional clearing agent xylene was not recommended to apply on large tissues because of shrinking and hardening tissues after long-time clearing^16^. When treating large samples, it was easy to make inside and outside these samples unevenly harden and shrink. Trials on embedding large tissues were few. Only one report mentioned it^17^. They embedded a whole human brain with FFPE in their study, but no protocol was shown. Thus, there was no detailed and standard protocol to guide how to embed large tissues with FFPE.

In this study, we presented a detailed protocol to embed large tissues with FFPE, which could be employed for semi-thin section and H&E staining. We embedded the mouse, rat, rabbit and macaque hemisphere and acquired the structural information. To the best of our knowledge, this was the first report that provided a detailed protocol for various sample sizes with a systematic evaluation and troubleshooting of the embedding quality.

## Materials and methods

### Animals

Ideally, the tissue being analyzed should be less than 60 cm^3^. So far, we had confirmed good imaging performance with C57/BL (The Jackson Laboratory), rat (Shanghai Model organisms), rabbit (Shanghai Model organisms) and macaques (Kunming Institute of zoology). All animals were cared for and treated humanely in accordance with governmental and institutional regulations regarding the use of animals for research purposes. Animal experiments must be performed in accordance with governmental and institutional regulations regarding the use of animals for research purposes. All animal experiments and housing conditions in this manuscript were approved by the Institutional Animal Ethics Committee of the Huazhong University of Science and Technology, and all animals were cared for and treated humanely in accordance with the institutional guidelines for experiments using animals.

### Paraffin embedding

The protocols were described in Table 3.

### Sectioning, staining and imaging

Tissue blocks were sectioned at 4–5 μm on a standard rotary microtome (Leica, Germany). These sections were collected and then stained by HE. The stained sections were mounted and examined microscopically (Nikon, NI-E).

## Results

### Optimization of paraffin embedding method

Clearing was an important step in the preparation of histological sections, aiming to remove alcohol and other dehydrating agents from tissues prior to infiltration of the paraffin wax. Xylene cleared tissues rapidly, and rendered transparency, facilitating clearing endpoint determination, this made it to be widely used in routine histopathological techniques^16^. However, xylene was not recommended to apply on large tissues because of shrinking and hardening tissues after long-time clearing. Moreover, it was an environmental hazard and highly toxic to humans. Therefore, we need to find a substitute for xylene if we wanted to embed large tissues. Various xylene-substitutes had been commercially developed to avoid the most obvious disadvantages of xylene^16,18–20^, including esters, aromatic, naphthenic, higher aliphatic hydrocarbons, terpene-based extracts and chlorinated hydrocarbons. We tested three xylene substitutions and compared them with xylene. Their advantages and disadvantages as clearing agents were presented (Tables 1 and 2). Although they were toxic, *n*-butyl alcohol and chloroform were still selected because of leaving tissues less hard. They were commonly used to clear large tissues based on the experimental accumulation of predecessors^17^, Therefore, they could be used as a compromise if we had no better choice. Considering synthetically, we deemed that using Histo-Clear II was better. There were four advantages. First, it left tissue less hard and brittle than xylene. Second, the penetrate rate was almost equivalent to xylene. Therefore, it could be used to instead of xylene without significant changes in experimental procedures. Third, this agent was non-toxic, non-volatile and odorless, as it was a mixture of aliphatic hydrocarbons and d-limonene. Finally, it could be biodegradable, which greatly reduces the cost of waste liquid treatment. Taking all of these advantages into account, we identified Histo-Clear II as a clearing agent in large tissues paraffin-embedding processing.

**Table 1:** Physical properties of clearing agents.

Physical properties	Xylene	*n*-butyl alcohol	Chloroform	Histo-clear II
Viscosity (mPa s)^a^	1	0	1	1
Volatility^b^	1	0	2	0
Cost^c^	1	0	1	2
Odor	Unpleasant	Unpleasant	Odorless	Pleasant
Health hazards	Yes	Yes	Yes	No
Disposal	Not recyclable	Not recyclable	Not recyclable	Recyclable

^a^Viscosity scoring: score-0 = less viscous than xylene, score-1 = equivalent to xylene.

^b^Volatility scoring: score-0 = less volatile than xylene, score-2 = more volatile than xylene.

^c^Cost comparison per 1000 ml of xylene: score-0 = economical compared to xylene, score-1 = approximate to xylene, score-0 = costlier than xylene.

**Table 2:** Comparison of gross changes in tissues cleared with different clearing agents.

Gross changes	Xylene	*n*-butyl alcohol	Chloroform	Histo-clear II
Translucency	1	0	0	0
Rigidity	1	1	1	1
Shrinkage	1	1	2	2
Section cutting	1	1	2	2

Score-0 = inferior to xylene, score-1 = equivalent to xylene, score-2 = superior to xylene.

**Figure 1:**
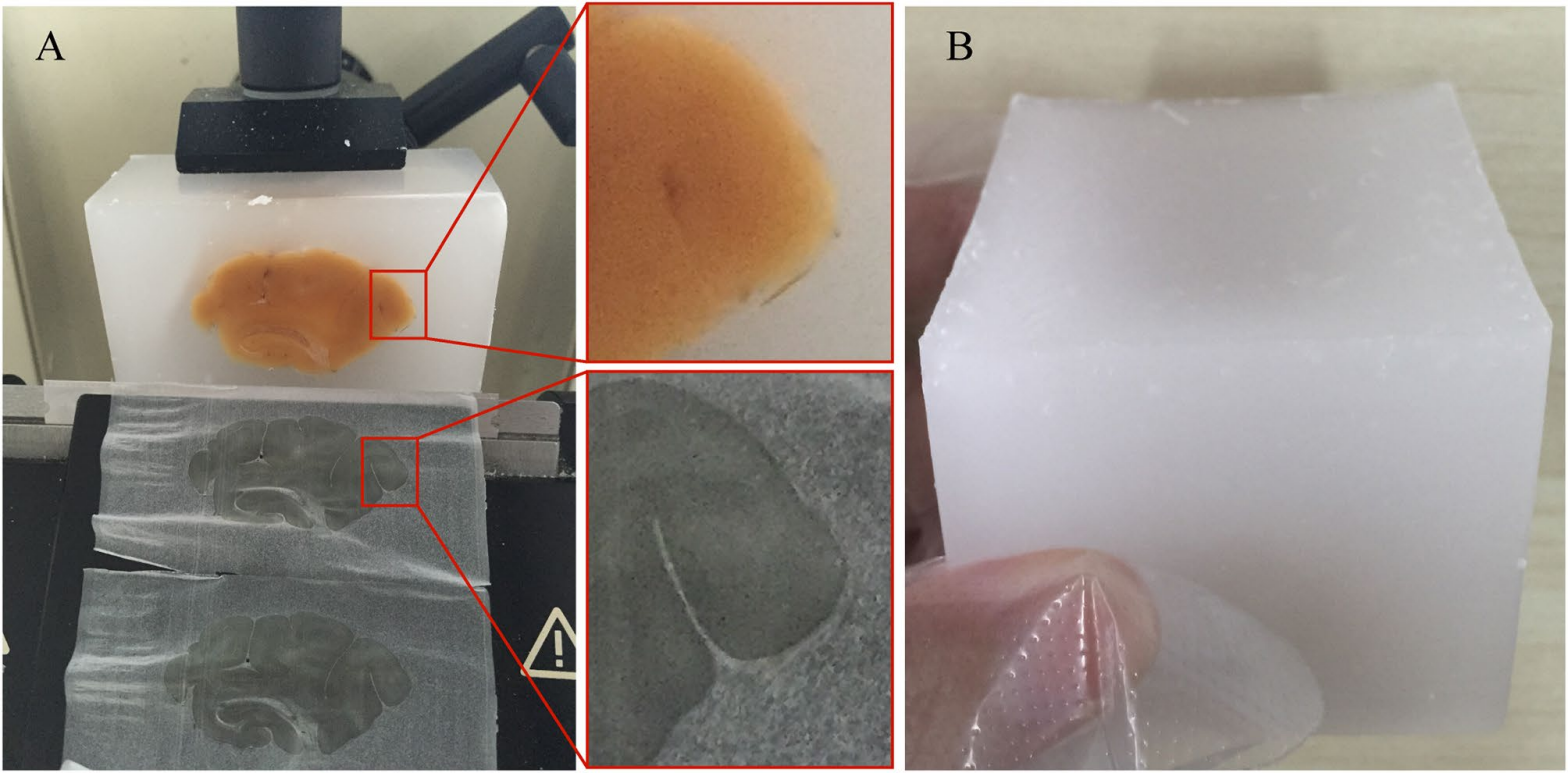
The effect of optimized process. (**A**) Samples when using the improved embedding process. (**B**) Normal embedded samples when using the silicon mold and improved embedding process.

**Figure 2:**
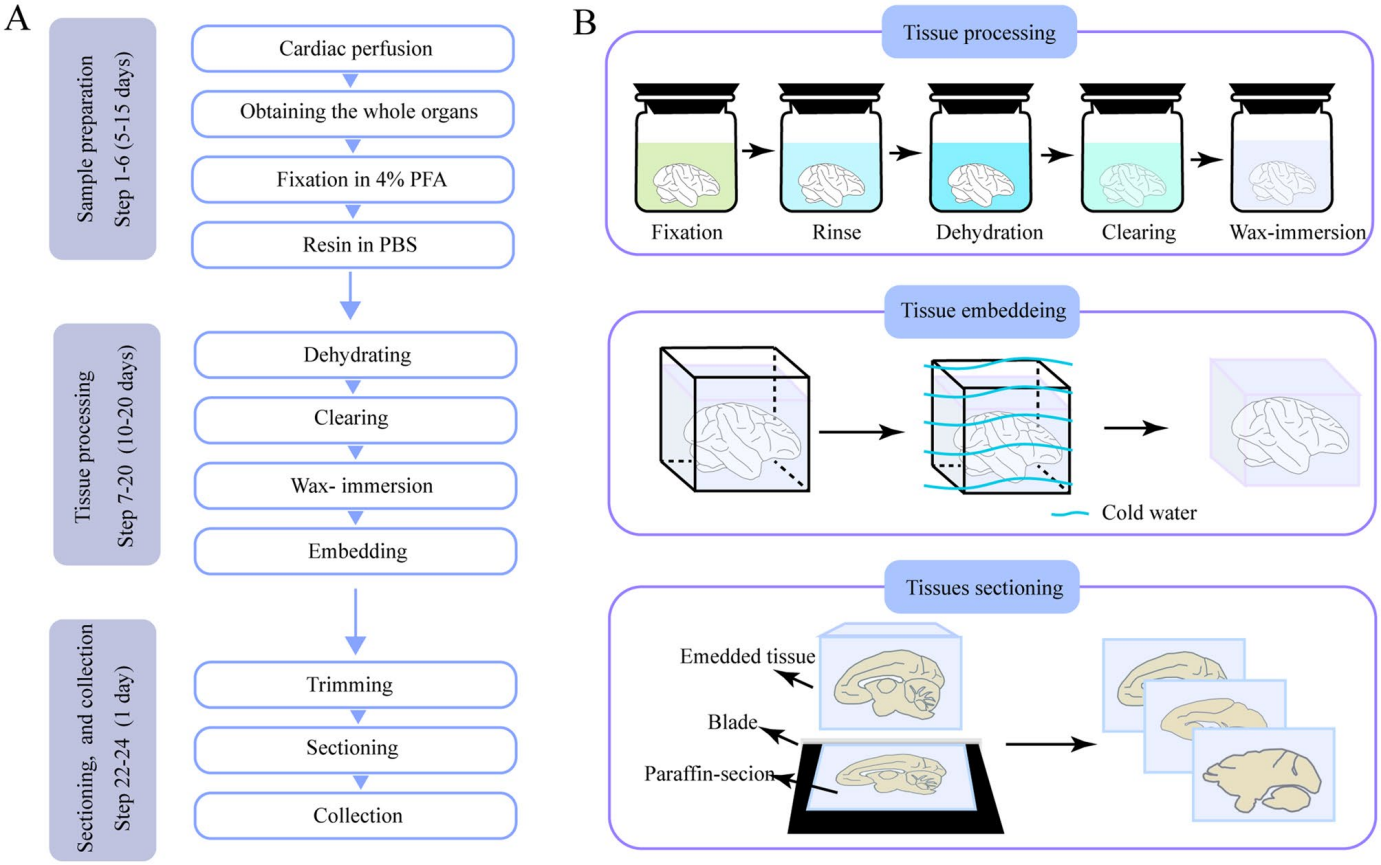
Schematic diagram for the entire experimental procedure. (**A**) Flowchart for the all steps of the protocol. (**B**) Schematic diagram illustrating the procedure for embedding large organs (take the macaque hemisphere as example).

Paraffin wax was melted in constant temperature oven at 65 °C and preserved at 60 °C after completely melted. After clearing, we put the specimen into paraffin wax. To remove as much clearing agent as possible, changing new paraffin at least 3 times. There were two troubles only for embedding large tissue, instead for small tissues. One was control of the solidified speed. Fast solidification resulted in the rupture of the paraffin wax block (Fig. S1A). Slow solidification affected sectioning of paraffin and resulted out a badly dented surface of the paraffin block (Fig. S1B). Another one was that there were lots of bubbles in the paraffin wax block and where the tissue connects to the paraffin (Figs. S1C and S1D). To solve these troubles, we adjusted embedding process and designed a mold. In this protocol, embedding process included three steps. First, we poured the paraffin wax into the mold before embedding and stored the mold for at least 12 h at 60 °C. This step can enable enough time for broken of these small bubbles that produced by pouring liquid paraffin. Second, we moved the specimen into the mold at least 4 h in advance at 60 °C. During this period, we shocked slightly the specimen every 2 h. Third, we removed the mold from the oven and waited until the surface of paraffin had solidified. Then, we carefully put the mold into water (about 10 °C). This adjusted process avoided production of bubbles to make more close connection between tissues and paraffin (Fig. 1A). Based on our adjusted embedding process, the mold should be heat resistant. Initially, the mold was made of aluminum alloy. However, we found the paraffin close to the mold walls solidified rapidly due to great thermal conductivity of aluminum alloy. In contrast, the paraffin at the center of the mold cooled very slowly. These speed differences resulted in a cavity on the surface of the paraffin block (Fig. S1E). Here, we designed a silicon mold, which has a poor thermal conductivity, to avoid questions mentioned above (Fig. 1B). The silicon bond was heat resistant, making it suitable for stored in high temperature. Meanwhile, samples could be removed from the silicone mold easily due to its excellent elasticity. Based on the above findings, we developed the optimized paraffin-embedding method (Fig. 2 and Table 3), which was suitable for large volume brains.

**Table 3:** Paraffin processing schedules.

Regent	Mouse brain (0.4 cm^3^)	Rat brain (1.6 cm^3^)	Rabbit brain (10 cm^3^)	Macaque hemisphere (60 cm^3^)
4% paraformaldehyde (PFA)	12 h	24 h	3 × 24 h	4 × 48 h
0.01 M phosphate buffer (PBS)	12 h	24 h	3 × 24 h	5 × 24 h
50% (vol/vol) alcohol	3 × 1 h	2 × 3 h	3 × 4 h	2 × 12 h
70% (vol/vol) alcohol	3 × 1 h	2 × 3 h	3 × 4 h	2 × 12 h
80% (vol/vol) alcohol	3 × 1 h	2 × 3 h	3 × 4 h	2 × 12 h
95% (vol/vol) alcohol	2 × 1 h	2 × 3 h	3 × 4 h	2 × 12 h
100% (vol/vol) alcohol	3 × 1 h	3 × 2 h	3 × 4 h	3 × 24 h
50% (vol/vol) Histo-clear II	1 h	2 × 1 h	3 × 3 h	2 × 12 h
100% (vol/vol) Histo-clear II	≥ 45 min	≥ 1 h	≥ 2 h	≥ 12 h
Paraffin-immersion	3 × 4 h	3 × 8 h	4 × 12 h	8 × 24 h
Embedding	2–3 h	2–3 h	6 h	Overnight

### Applications on large volume tissues

**Figure 3:**
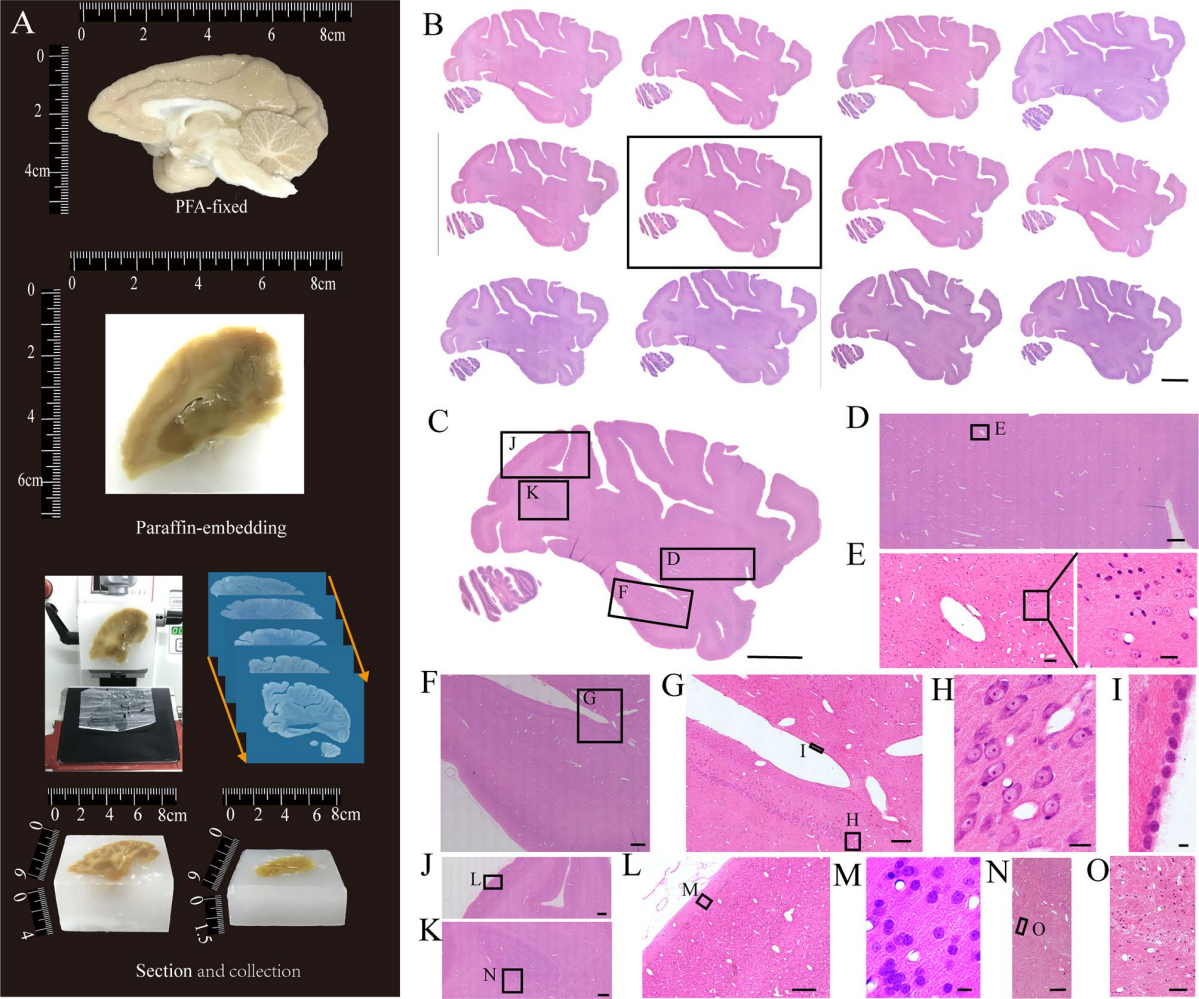
Slices from an intact macaque hemisphere embedded by paraffin, stained with H&E. (**A**) Histological sectioning. (**B**) The serial sagittal plane slices from the macaque hemisphere. All the sections were sectioned at the coronal plane for 5 μm. (**C**) Magnification of the box in (**B**). (**D**) Magnification of the box labeled D in (**C**). (**E**) Magnification of the box labeled E in (**D**). (**F**) Magnification of the box labeled F in (**C**). (**G**) Magnification of the box labeled G in (**F**). (**I**, **H**) Magnification of the boxes labeled I and H in (**G**). (**J**, **K**) Magnification of the boxes labeled J and K in (**C**). (**L**) Magnification of the box labeled L in (**K**). (**M**) Magnification of the box labeled M in (**L**). (**N**) Magnification of the box labeled N in (**K**). (**O**) Magnification of the box labeled O in (**N**). Scale bars, (**B**, **C**) 1 cm; (**D**) 1 mm; (**E**) 100 μm, black box, 20 μm; (**F**) 1 mm; (**G**) 200 μm; (**H**) 20 μm; (**I**) 5 μm; (**J**, **K**) 1 mm; (**L**) 200 μm; (**M**) 10 μm; (**N**) 200 μm; and (**O**) 50 μm.

We had used this improved method to successfully the macaque hemisphere (Fig. 3). Intact and continuous sections were collected and stained by H&E (Fig. 3A, B). To evaluate preservation of structural morphology in brains, we could assess the structure of brain areas and distribution of cells (Fig. 3C). The contrast between the blue-purple nucleus and the pink background was sharp, and the cell bodies contour were well defined (Fig. 3D–O).

**Figure 4:**
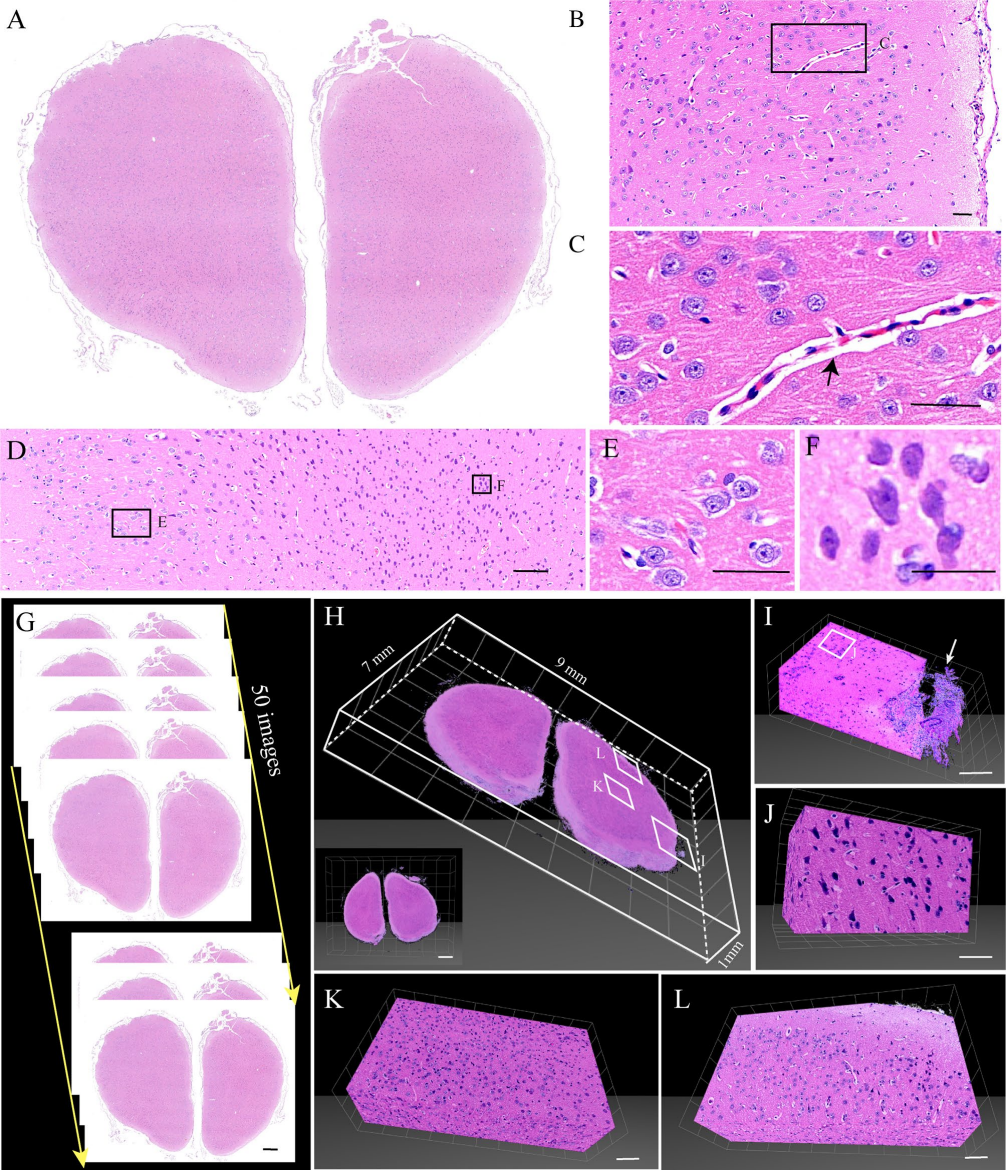
Slices from an intact rabbit brain embedded by paraffin, stained with H&E. (**A**) The coronal plane slices from the rabbit brain. (**B**) Magnification of (**A**). (**C**) Magnification of (**B**). Blood vessels indicated by the black arrow. (**D**) Magnification of (**A**). (**E**, **F**) Magnification of (**D**). (**G**) The coronal plane slices from the rabbit brain. All the sections were sectioned at the coronal plane for 4 μm, and we collected serial 50 images. (**H**) 3D reconstructed images of (**G**). (**I**) Magnification of (**H**). The meninx indicated by the white arrow. (**J**) Magnification of (**I**). (**K**, **L**) Magnification of (**H**). Scale bars, (**A**) 500 μm; (**B**, **C**) 50 μm; (**D**) 100 μm; (**E**) 50 μm; (**F**) 25 μm; (**G**) 500 μm; (**H**) white grid, 1 mm; (**I**) white grid, 100 μm; (**J**) white grid, 50 μm; (**K**, **L**) white grid, 100 μm.

Notably, the entire tissue embedding and the perfect sectioning performance and combination with H&E, making it potential in combination with 3D reconstruction technologies. We took the rabbit brains as example and reconstructed the 3D images of H&E (Fig. 4). The results showed that paraffin sections were complete without any small crack, and the cell bodies contour were well defined (Fig. 4A–F). 50 consecutive sections of prefrontal cortex were selected and then reconstructed to 3D images (Fig. 4G–L). Therefore, based on natural 3D boundary information it was possible to classify the tumor stage for judging the tumor invasion, which could be applicable to offer new insights into the study of neuroscience. These intact and continuous sections indicated our method was compatible with large volume brains.

Paraffin embedding method we developed here was a simple, efficient and reproducible method and the procedure could be performed using equipment usually used in a typical biology laboratory. It broke the volume limitation of traditional FFPE and could be useful for bioscience studies.

## Discussion

### Quality evaluation and troubleshooting

To achieve the best embedding quality, careful preparation of the samples was crucial. The quality of paraffin-embedded samples could be examined when sectioned. After the paraffin solidification, there should be no air bubbles in the paraffin block and the connection between samples and paraffin block (Fig. 1A). Also, impurities such as white floccus should not be appeared. White floccus indicated excessive clearing agent in paraffin (Fig. S1F). We should increase the frequency of replacement of paraffin or change new paraffin. When a cavity appeared in the center of a sample (Fig. S1G), it indicated that the sample was not completely dehydrated. If a white circle appeared in the center of the tissue accompanying a strong odor of the clearing agent (Fig. S2), it indicated inadequate paraffin-immersion. We could move the sample to new melted paraffin and prolong the time of paraffin-immersion. This operation did not affect sectioning performance, but it was unfriendly to successional sections collection. When sectioning difficultly with slices completely or partly crumble, packing precool iron sheet on the surface of paraffin block for 10–15 s can greatly help section. We found this remedial action could completely rescue serious consequences caused by excessive dehydration or clearing. In order to popularize this method, we summarized the problems and solutions during paraffin embedding process (Table S1).

### Applications and advantages of the method

The most significant advantage was that our method provided the ability to produce whole-mount of brains with different size and other organs (Table 3, Figs. S3 and S4). It broke the limitations for analyzing large samples. Tissues available in our approach were about a factor of 1–80 larger than in conventional methods (volume, from 0.4 to 60 cm^3^)^21^. Compared with previous studies^21,22^, this no-dissecting approach enabled sample integrity and unambiguously captures tissue orientation. It markedly reduced tissue loss or fragmentation and conformational change caused by dissection. All of these characteristics provided more accurate and meaningful structure measurements. The present method could process the entire specimens for continuous and intact sections, which could reduce distortion. On the other hand, compared with resin embedding and agar embedding, embedding method commonly used in 3D imaging technology, the embedding time of our method was completely acceptable. Take mouse brains as example, it about took 1.5 days by using agar embedding, 6 days by using resin embedding^8^ and 3 days by using our method. Moreover, this method was low-cost, that consists of sequential solution steps and only requires only a few minutes of actual work interspersed between incubation times, while can be done without special equipment. It can be used to study tissue 3D architecture and neuronal morphology in the large-volume tissue such as the whole primate brain.

## Supplementary information

Supplementary file1

## Data Availability

All data needed to evaluate the conclusions in the paper were present in the paper and/or the Supplementary Materials. Additional data related to this paper may be requested from the authors.
