# Supplementary material for: Paraffin-embedding for large volume bio-tissue
Source: Sci Rep. 2020 Jul 28;10:12639. doi: 10.1038/s41598-020-68876-5 (PMC7387479; doi:10.1038/s41598-020-68876-5)
Supplement: Supplementary file 1 — Supplementary file1 [file 41598_2020_68876_MOESM1_ESM.docx]

**Paraffin-embedding for large volume bio-tissue**

Ouyang Zhanmu^1,2^, Xiaoying Yang^1,2^, Hui Gong^1,2,3^, and Xiangning Li^1,2,3^*

^1^Britton Chance Center for Biomedical Photonics, Wuhan National Laboratory for Optoelectronics-Huazhong University of Science and Technology, Wuhan, Hubei 430074, China

^2^MoE Key Laboratory for Biomedical Photonics, Collaborative Innovation Center for Biomedical Engineering, School of Engineering Sciences, Huazhong University of Science and Technology, Wuhan, Hubei 430074, China

^3^HUST-Suzhou Institute for Brainsmatics, Suzhou 215125, China

*Corresponding author. Email: lixiangning@mail.hust.edu.cn


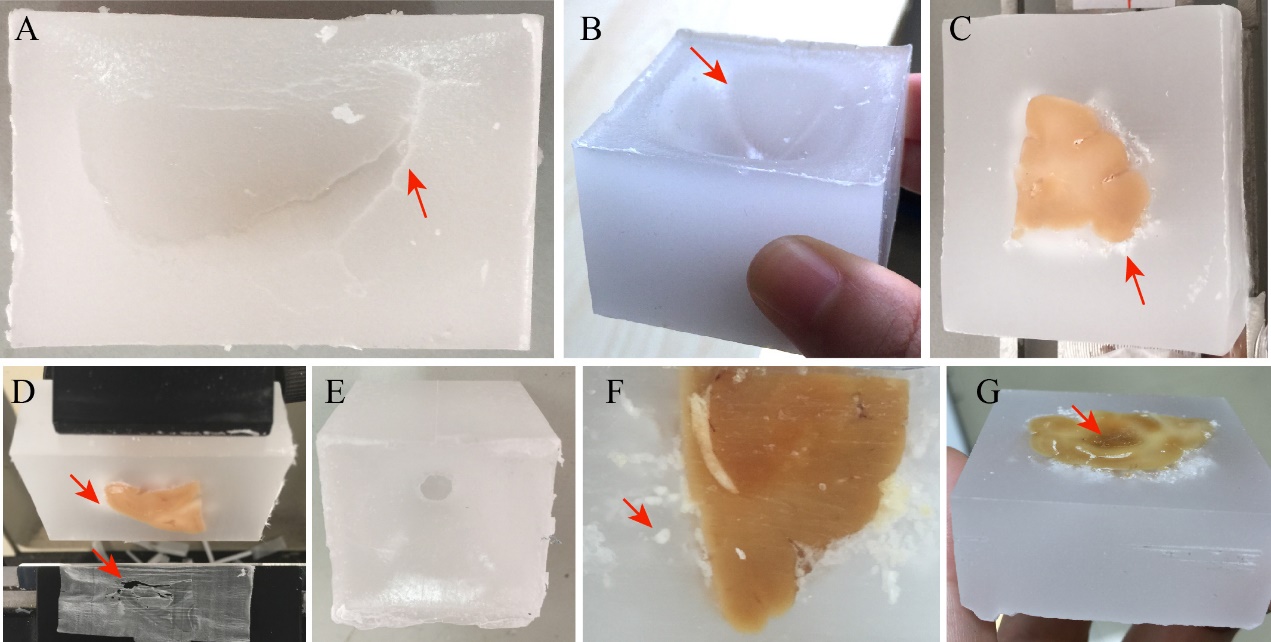


**Supplementary Fig 1. Some problems during paraffin embedding. (A)** Rapid solidification resulted in the rupture of the paraffin block, which was indicated by the red arrow. **(B)** Slow solidification resulted in a badly dented surface of the paraffin block, which was indicated by the red arrow. **(C and D)** Traditional embedding method generated lots of bubbles between paraffin block and tissues. These bubbles were indicated by the red arrow. **(E)** A cavity appeared on the surface of the paraffin block when using metal mold. The cavity was indicated by the red arrow. **(F)** Inadequate dehydration resulted in a cavity in the center of the sample. **(G)** Excessive clearing agent resulted in lots of white floccus. The white floccus was indicated by the red arrow.


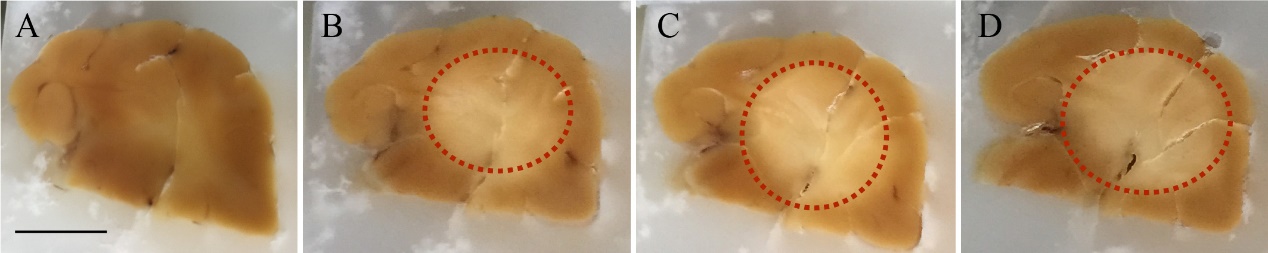


**Supplementary Fig 2. Samples with insufficient-immersion.** The part of samples with inadequate paraffin-immersion was indicated by a red circle. Scale bar, 1 cm.


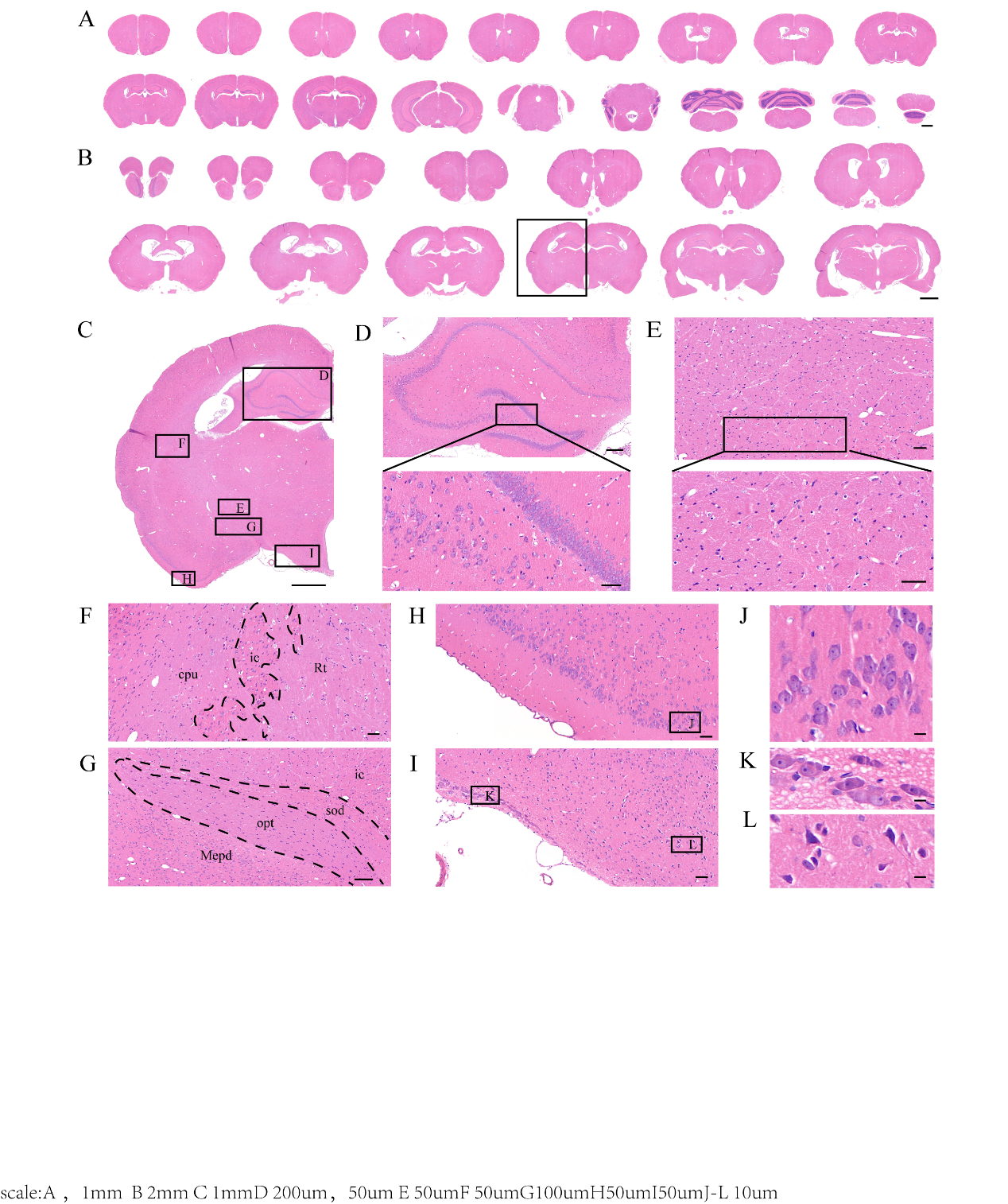
 **Supplementary Fig 3. Slices from an intact mouse brain and rat brain embedded by paraffin, stained with H&E.** (**A**) The coronal plane slices from the C57BL/6 mouse brain. (**B**) The coronal plane slices from the SD rat brain. All the brains were sectioned at the coronal plane for 4 μm, and we selected mouse brain slices for every 500~1000 μm and rat brains sections for every 1.5~3mm from the prefrontal lobe to the epencephalon. (**C**) Magnification of (B). (**D-I**) Magnification of (C). (**J**) Magnification of (H). (**K and L**) Magnification of (I). cpu, caudate putamen; ic, internal capsule; Rt, reticular thalamic nucleus; sod, supraoptic decussation; opt, optic tract; Mepd, medial amygdaloid nucleus, posterodorsal part. Scale bars, (A and B) 2 mm; (C) 200 μm; (D) 200 μm, black box, 50 μm; (E) 50 μm, black box, 50 μm; (F) 50 μm; (G) 100 μm; (H-I) 50 μm; and (J-L) 10 μm.


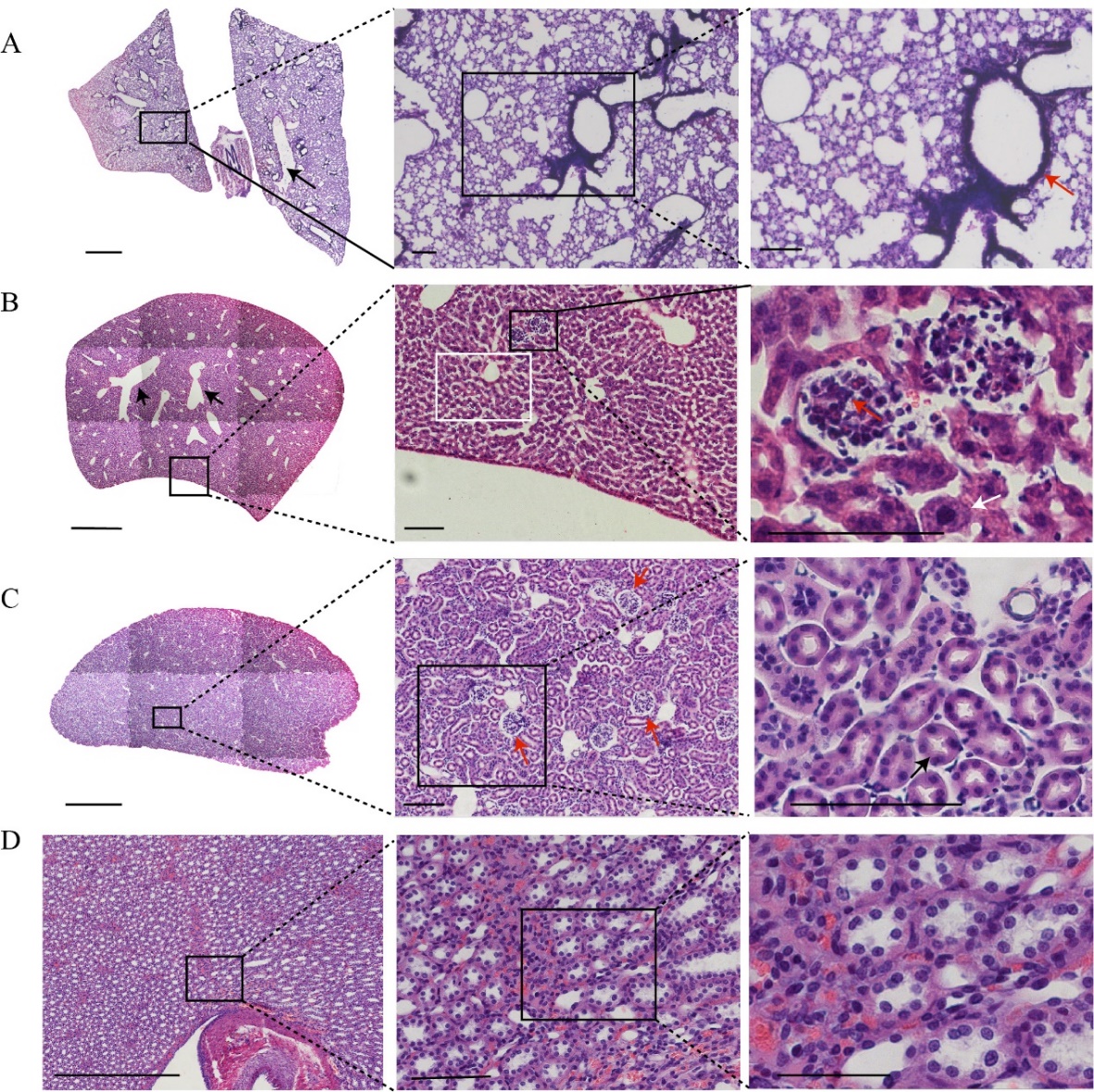


**Supplementary Fig 4 Morphological preservation of the lung, the liver and the kidney embedded by paraffin.** **(A)** Images of the mouse lung. The trachea was indicated by the black arrow, and the alveoli was indicated by the red arrow. **(B)** Images of the mouse liver. Blood vessles were indicated by black arrows, and the inflammatory factors were indicated by red arrows. The structure of liver plate was indicated by white rectangle, and the liver cell was indicated by the white arrow. **(C)** Images of the mouse kidney. The glomeruli were indicated by red arrows, and the renal tubules were indicated by black arrows. **(D)** Images of the rabbit kidney. Scale bar, 1 mm, black box, 100 μm.

**Supplementary Table 1 Troubleshooting table**

| Problem | Possible reason | Solution |
| --- | --- | --- |
| Morphological changes | PFA post-fixation time is too short | Prolong the post-fixation time to 15 d |
| Sectioning difficultly because of hardness | Excessive dehydration | Shorten the time of absolute EtOH to 48-60 h |
| A cavity in the center of embedded tissues | Dehydration time is too short | Prolong the dehydrating time |
| Murky clearing agent | Incomplete dehydration | Dehydration in absolute EtOH again |
| Sectioning difficultly because of hardness | Excessive clearing | Shorten clearing time |
| Sectioning difficultly because of softness | Incomplete clearing | Prolong clearing time |
| A white circle in the embedded tissues | Incomplete paraffin wax immersion | Prolong paraffin wax immersion time |
| A strong odor of the clearing agent | Excessive clearing agent in paraffin wax | Increase the frequency of replacement of paraffin wax |
| White floccus in paraffin block | Excessive clearing agent in paraffin wax | Chang new paraffin wax or increase the frequency of replacement of paraffin wax |
| Bubbles appear in paraffin block | Inadequate time to discharge the bubbles | Pour melted paraffin wax into the silicon mold 24 h in advance |
| Bubbles appear in the connection between paraffin block and tissues | Inadequate shaking frequency or small shaking amplitude | Increase shaking frequency or waggle the tissue with tweezers |
| Rough surface of paraffin block | Inadequate time for solidification of surface of paraffin wax | Wait for more time |
| Splits appear in the paraffin block | Low temperature of water | Raise the temperature of water |
| Section difficultly with slices completely or partly crumble | Excessive dehydration or clearing | Pack precool iron sheet on the surface of paraffin block for 10-15s can help section; Shorten dehydrating or clearing time |
| Vertical scratch on sections | A gap in the blade | Chang a new blade |
| Rolling up sections | Sections are too thick or | Thickness of 10-20μm are recommended for large tissues |
| Folding sections | Sections are too thin or paraffin wax is soft | Thickness of 10-20μm are recommended for large tissues or change high melt point paraffin wax. |
| Tissues and paraffin are separated | High temperature of water | Decrease the temperature of water |
